# Retrieval Practice and Word Learning in Children With Specific Language Impairment and Their Typically Developing Peers

**DOI:** 10.1044/2020_JSLHR-20-00006

**Published:** 2020-10-15

**Authors:** Laurence B. Leonard, Patricia Deevy

**Affiliations:** a Purdue University, West Lafayette, IN

## Abstract

**Purpose:**

In this article, we review the role of retrieval practice on the word learning and retention of children with specific language impairment (SLI).

**Method:**

Following a brief review of earlier findings on word learning in children with SLI and the assumptions behind retrieval practice, four experiments are described that compared novel words learned in a repeated spaced retrieval condition and those learned in either a repeated study condition or a repeated immediate retrieval condition. Preschool-age children with SLI and their same-age peers with typical language development were the participants in all experiments. The effects of repeated spaced retrieval were assessed through measures of recall of word form and meaning and, receptively, through both picture-pointing and electrophysiological measures.

**Results:**

Repeated spaced retrieval resulted in greater recall of word form and meaning across the experiments. This advantage was seen not only for word–picture pairs used during the learning period but also when generalization of the word to new pictures was required. Receptive testing through picture pointing showed similar results, though in some experiments, ceiling effects rendered this measure less sensitive to differences. An alternative receptive measure—the N400 elicited during picture–word mismatches—showed evidence at the neural level favoring repeated spaced retrieval. The advantages of repeated spaced retrieval were seen in both children with SLI and their typically developing age mates.

**Conclusion:**

Future efforts are warranted to refine and extend the experiments reviewed here. If these efforts prove successful, procedures that incorporate repeated spaced retrieval into more naturalistic clinical and educational activities might be an appropriate next step.

**Presentation Video:**

https://doi.org/10.23641/asha.13063730

There is a common assumption that “practice” means “learning by doing.” Whether the goal is playing a musical instrument, skating backward, or throwing a pot, we recognize that real gains cannot be won through passive study alone. In this review article, we apply this concept to word learning. Our basic tenet is that, through the practice of retrieving words during the learning process, our retention of these words will be significantly enhanced. Studying the words without such retrieval practice will not be as effective, especially for long-term retention. The studies reviewed here will focus on one group of children for whom retrieval practice may be especially beneficial. These are children with “specific language impairment” (SLI)—a group of children whose language problems include weaknesses in learning words. Following a brief review of these children's word learning difficulties, we discuss the basic characteristics of retrieval practice and how these have been applied in studies of word learning by children with SLI.

## Word Learning and SLI

Children with SLI (also referred to as children with “developmental language disorder” or DLD in the recent literature) have a long-standing deficit in language ability that cannot be attributed to hearing impairment, intellectual disability, neurological damage, or autism spectrum disorder. Weaknesses in cognitive and motor areas are sometimes seen in these children but are not of the magnitude or nature to be the cause of the language disorder. Significant deficits in language ability are present from childhood, and in spite of gains due to maturation and treatment, the problems with language persist into adulthood (Law et al., 2008; Rice et al., 2010; Tomblin et al., 2003).

During the preschool years, the most salient symptoms of SLI usually fall in the area of morphosyntax, where grammatical omissions and poorly constructed sentences are readily apparent. Less obvious but easily detected through systematic study are the problems that these children experience with word learning. These children exhibit a wide variety of vocabulary-related deficits (e.g., Kail & Leonard, 1986; Leonard et al., 1983; McGregor et al., 2002). They have fewer words in their vocabulary, and they possess a relatively shallow understanding of many of the words they do use. This is not a short-term problem; these children fall further behind their peers over time (Rice & Hoffman, 2015).

To acquire a better picture of the word learning process in children with SLI, some investigators have studied these children's acquisition of novel words presented to them under controlled experimental conditions. The general picture is that these children require more exposures to these novel words than do their typical peers to reach the same success level (e.g., Alt et al., 2004; Gray et al., 2014); the weakness is seen in both comprehension and production (Kan & Windsor, 2010); and this reduced ability is seen across novel words representing nouns, verbs, and adjectives (Oetting et al., 1995).

The word learning deficits observed in children with SLI stand in contrast to the age-appropriate scores that many of these children earn on standardized tests of vocabulary ability. There are several interrelated reasons for such an apparent discrepancy. First, children with SLI are often included in studies based on their low scores on tests with acceptable sensitivity and specificity. These tests tend to emphasize morphosyntax, such as the Structured Photographic Expressive Language Test–Preschool 2 (SPELT-P 2; Dawson et al., 2005) and the Rice/Wexler Test of Early Grammatical Impairment (Rice & Wexler, 2001). Tests that focus on vocabulary have been shown to have inadequate sensitivity and specificity (Gray et al., 1999; Spaulding et al., 2013). Therefore, they are usually administered to provide only descriptive information about the children. Often, the resulting scores on these tests do not fall significantly below age-level expectations.

However, this is not to say that children with SLI score as high on these tests as children with typical language development from the same communities. For example, Gray et al. (1999) found that the mean standard scores for their SLI group on two expressive vocabulary tests and two receptive vocabulary tests were at age-appropriate levels, yet these scores were significantly lower than the scores of their typically developing (TD) peers on all four tests. McGregor et al. (2012) found that the scores of 13 of 14 children with SLI on a test of receptive vocabulary were well within 1 *SD* of the standardization mean, yet their scores nevertheless lagged significantly behind those of their peers with typical language development.

Given these group differences, one might argue that the “low normal” scores of children with SLI on standardized tests of vocabulary are somewhat misleading. These tests usually focus more on breadth than on depth of vocabulary, and in the case of receptive tests, they often rely on recognition. Furthermore, as tests of accumulated vocabulary knowledge, they shed very little light on the process by which children learn new words. Indeed, some studies have found no correlation between children's learning of novel words and their vocabulary test scores (e.g., Gray, 2003).

Several studies have begun the work of isolating the particular aspects of word learning that are most problematic for individuals with SLI. Two distinctions pertinent to word learning that have emerged as especially relevant are the distinctions between word form and meaning and between encoding and long-term retention. Word form refers to the phonetic form of the word itself, such as /sӕksǝfon/ (*saxophone*), separate from its meaning (a musical instrument). Although children and adults with SLI (or those given the label DLD) can have difficulty with meaning, learning word forms appears to be a bigger obstacle (Gray, 2004; McGregor et al., 2013). Likewise, word encoding—the process of putting the word in a storable form—appears to be a taller hurdle than retaining the word over the long term (McGregor et al., 2017). Much less is known about the word retrieval process in SLI and how it interacts with word forms and meanings and the degree to which it plays a role in long-term retention. The studies we review in this article sought to answer these questions.

## The Benefits of Retrieval Practice

Scholars have long observed that when we test ourselves as we study new material, our learning is facilitated. Testing is not a neutral event representing only an assessment of what we have already learned. It is itself a type of learning. This observation has been made for many years, perhaps beginning with Ebbinghaus (1885, translated in *Annals of Neurosciences,* 2013). What may be the first empirical study of this phenomenon dates back to Edwina Eunice Abbott's master's thesis (Abbott, 1909a), published later the same year in *Psychological Monographs* (Abbott, 1909b). Abbott set the stage for her work with the following call for scientific rigor:

No one who has had experience in memorizing or in watching others memorize can have failed to observe the tendency of the average person when he is reading, to momentarily turn away from the material before him and to repeat it to himself without external aid…. The fact that this tendency is to be observed, however, does not justify us in believing without further evidence that recall is a desirable and helpful factor in the learning process. (Abbott, 1909a, p. 1)

Indeed, through her systematic study, Abbott (1909a, 1990b) did find that this type of activity benefited learning.

In recent years, there has been a resurgence of research on the effects of retrieval practice. We highlight here two of the factors shown to be important in this line of work, as these formed the basis for our research on word learning by children with SLI.

### Repeated and Spaced Retrieval

One factor is the frequency with which retrieval is attempted or, in the parlance of experimental psychology, the degree to which retrieval trials are inserted during the study period. When research participants attempt to retrieve information frequently over the course of the learning period, their retention of that material is superior to the retention seen when the participants repeatedly study material without any retrieval activity. As shown by a meta-analysis of 159 studies by Rowland (2014), this finding holds across a wide range of experimental manipulations. The material to be learned in these studies has varied widely, from word lists to science concepts. Research with school-age children has had more of an applied focus, in which participants learned educationally relevant material (see review in Fazio & Marsh, 2019).

A second factor proving important is the spacing between retrieval attempts. Spacing can occur in the temporal sense, but in many experiments, spacing is created by inserting other items between the previous study trial of the target item and its subsequent retrieval. The degree of spacing must be sufficient for retrieval to be “effortful” but narrow enough to prevent forgetting. Material learned through (repeated) spaced retrieval shows better long-term retention than material learned through (repeated) immediate retrieval (IR; e.g., Karpicke & Roediger, 2007). Although research with adults suggests that equally spaced retrieval trials are sufficient to show the advantage over IR, studies with children sometimes use an expanding retrieval schedule in which the early retrieval trials appear immediately after a study period and the spacing is gradually increased (Fazio & Marsh, 2019). Note that in studies comparing spaced retrieval and IR, the two conditions are matched not only for the number of times the material is presented (which is the case in repeated retrieval vs. repeated study [RS] comparisons) but also in the number of retrieval opportunities provided.

### The Episodic Context Account

Although “effortful” is an appropriate term for the learning pattern seen with repeated spaced retrieval (RSR), it merely describes the process; it does not explain it. There have been several accounts of the mechanisms that might be responsible for producing the benefits seen with this type of retrieval. Our own work has operated within the framework of the episodic context account of Karpicke and his colleagues (Karpicke, 2017; Karpicke et al., 2014; Karpicke & Zaromb, 2010; Lehman et al., 2014). Because we did not compare competing accounts, we do not claim our findings uniquely support the episodic context account. However, our findings of the advantages conferred by RSR relative to repeated IR and RS are compatible with this approach.

A key assumption of the episodic context account is that features of the context become associated with the to-be-learned items during encoding. During retrieval, these contextual features are reinstated during memory search. Each time retrieval is successful, the contextual representation is updated to include the features associated with the most recent retrieval. The composite of features built through successive retrieval events allows the target item to become more distinct relative to its competitors, thereby aiding recall. This account therefore emphasizes the value of “repeated” retrieval. However, its assumptions also provide the means of explaining the advantages of “spaced” retrieval. When other items intervene between a target item's previous study trial and its retrieval, the set of contextual features associated with each retrieval will create a more distinct representation than will the set associated with IR, given the greater changes in context resulting from increased time and the appearance of intervening items.

In daily life, it is easy to imagine how context changes with multiple, spaced attempts at retrieval. We can recall a statement made in a classroom by a professor after we have left the classroom and are sitting in the library and then again when we are at home. Both the physical setting and time have changed during each of these instances of retrieving the professor's statement. In tightly controlled experimental tasks, changes in context are more subtle. However, through some clever experimental procedures, several studies have produced results that can be unambiguously attributed to contextual effects. For example, Whiffen and Karpicke (2017) had participants study two word lists in succession, separated by a short distractor task. Then, the words were presented in a single, mixed list. One group of participants simply restudied the material, while the other group was asked to try to remember which list each word appeared in. During final testing, the group that tried to retrieve the specific list information had better recall of the words. For such differences to occur, the participants had to have associated the contextual information of list number with each word during encoding (without having been asked to do so); otherwise, this information would not have been available when they were asked to retrieve it after the words appeared in the single list with mixed order.

## The Contribution of Retrieval Practice to Children's Word Learning

Considering the growing literature on retrieval effects on children's learning, surprisingly few studies have used novel words as the material to be learned (see Fritz et al., 2007, for one of the few examples with TD child participants). To our knowledge, prior to 2019, only a single investigation of this type had been conducted with children with SLI (Chen & Liu, 2014). Another investigation examined retrieval effects in a group of young adult participants referred to as exhibiting DLD (McGregor et al., 2017). Both studies reported word learning advantages for retrieval.

The study by McGregor et al. (2017) serves as a good example. McGregor and her colleagues asked their participants to learn (a) a set of novel words with no retrieval during the learning period, (b) a set in which the participant had to recall the novel word after each study trial, and (c) a set in which retrieval was required but the experimenter provided the participant with the first syllable of the word. On a recall test 1 day after the learning period, the individuals with DLD showed recall on par with that of same-age peers with typical language for the words in the two retrieval conditions, but poorer recall than their peers for words learned through study with no retrieval practice.

## Retrieval Practice in SLI: Four Experiments

Based on the documented word learning difficulties of children with SLI and the extant literature on retrieval,our laboratory conducted a series of studies aimed at discovering the nature and potential benefits of retrieval pratice for these children. We review four of these studies here. We begin with a description of the research participants, as we applied similar selection criteria across the studies.

### Participants

Preschool-age children with SLI and children with typical language development of similar chronological age participated in each study. In each study, children averaged 5 years of age, with a range of 4;3–5;11 (years;months). Two of the children in the SLI group participated in Experiment 1 and, 1 year later, in Experiment 4. The children in Experiment 3 represented a subset of the participants in Experiment 2, as this experiment involved obtaining neural measures of the learning that occurred in Experiment 2. In all other cases, each child participated in only one of the experiments. The children with SLI met the now-traditional criteria for this designation. All children were either enrolled in a language intervention program or were scheduled for enrollment in such a program. They passed a hearing screening, had no history of neurological damage or disease, and fell in the “nonautistic” range on a screening for autism (Schopler et al., 2010). The children showed age-appropriate nonverbal IQs averaging between 98 and 101 across the studies. Most children were administered the Kaufman Assessment Battery for Children, Second Edition (Kaufman & Kaufman, 2004), though a few of the children in Experiment 1 were given the Primary Test of Nonverbal Intelligence (Ehrler & McGhee, 2008).

Our criteria for language followed a two-step process. All children were administered the SPELT-P 2. If they scored below the cutoff for acceptable sensitivity and specificity on this test (Greenslade et al., 2009), they were included. If children scored just above this cutoff score, they were required to score below the sensitivity/specificity cutoff on the finite verb morphology composite (Gladfelter & Leonard, 2013; Souto et al., 2014) and/or score below the 10th percentile on Developmental Sentence Scoring (Lee, 1974). Four of the 40 children in the SLI group undertook this additional testing to meet the selection criteria. The children in the TD group met the same nonlanguage criteria and scored well above the cutoff on the SPELT-P 2.

As noted earlier, standardized vocabulary tests do not fare well as selection criteria given their weak sensitivity and specificity. However, for descriptive purposes, we administered representative tests of this type. For Experiment 1, this was the Peabody Picture Vocabulary Test, Fourth Edition (PPVT-4; Dunn & Dunn, 2007). For the remaining experiments, both the PPVT-4 and the Expressive Vocabulary Test, Second Edition (EVT-2; Williams, 2007) were administered.

Table 1 provides a summary of the children's test scores in each experiment. Note that, as in previous studies, the children with SLI in our studies showed standardized vocabulary test scores at age-appropriate levels. In turn, their TD peers showed above-average scores. In all instances, the scores of the children with SLI proved to be significantly lower than the scores of the TD children serving in the comparison groups. For each experiment, the group difference on both the PPVT-4 and EVT-2 reflected very large effect sizes; Cohen's *d* ranged from 1.28 to 1.73.

**Table 1.** Mean standard scores (and standard deviations) on the standardized tests administered to the children with specific language impairment (SLI) and the children with typical language development (TD).

Experiment	Test	Group	
1		SLI (*n* = 10)	TD (*n* = 10)
	SPELT-P 2	74.70 (12.48)	118.90 (7.48)
	KABC-II or PTONI	108.40 (12.14)	121.60 (17.06)
	PPVT-4	97.67 (9.70)	115.20 (13.21)
2		SLI (*n* = 16)	TD (*n* = 16)
	SPELT-P 2	78.69 (9.41)	113.06 (9.17)
	KABC-II	101.88 (8.00)	115.81 (10.06)
	PPVT-4	103.44 (9.91)	121.06 (12.47)
	EVT-2	99.25 (7.99)	114.56 (9.79)
3		SLI (*n* = 14)	TD (*n* = 13)
	SPELT-P 2	78.57 (8.99)	114.08 (10.26)
	KABC-II	103.07 (9.14)	116.62 (11.33)
	PPVT-4	104.93 (8.88)	123.38 (13.14)
	EVT-2	100.29 (8.79)	115.54 (10.86)
4		SLI (*n* = 14)	TD (*n* = 13)
	SPELT-P 2	76.93 (15.78)	119.00 (8.03)
	KABC-II	99.21 (12.88)	114.31 (11.06)
	PPVT-4	102.57 (11.33)	118.62 (13.62)
	EVT-2	100.79 (9.25)	115.92 (8.16)

*Note.* The children in Experiment 3 were a subset of those participating in Experiment 2. SPELT-P 2 = Structured Photographic Expressive Language Test–Preschool 2; KABC-II = Kaufman Assessment Battery for Children, Second Edition; PTONI = Primary Test of Nonverbal Intelligence; PPVT-4 = Peabody Picture Vocabulary Test, Fourth Edition; EVT-2 = Expressive Vocabulary Test, Second Edition.

### Experiment 1: Repeated Retrieval Versus Repeated Study

Our first study in this area pitted repeated retrieval against RS (Leonard, Karpicke, et al., 2019). We asked if retrieval advantages could be seen in children with SLI at the preschool age, whether these advantages would apply to word form as well as meaning, and whether they could be observed not only immediately after learning but also 1 week later.

For this study, 10 children with SLI and 10 same-age TD peers participated. The children in both groups averaged age of 5;3. The children learned eight novel monosyllabic consonant–vowel–consonant words (e.g., /nɛp/, /paɪb/) from Storkel and Lee (2011). These served as the names of unusual plants and animals and are hereafter referred to as the “word forms.” We also created “meanings” for each novel word by associating each plant or animal with what it “liked,” such as birds or rain. The words were divided into two sets and were taught in succession, with 1 week separating the learning sessions for the sets. Within each set, two words were assigned to the repeated retrieval condition and two to the RS condition. Thus, the two learning conditions were compared through a within-participant design.

There were 16 study trials for each word, regardless of learning condition. In these trials, the child saw the picture of the plant or animal and heard (using /nɛp/ as the example) *This is a /nɛp/. It*'*s a /nɛp/. A /nɛp/ likes birds.* Words in the repeated retrieval condition also had 12 retrieval trials. In these trials, the child saw the picture and was asked *What*'*s this called? What do we call this?*—a request for word form. Once the child responded, the picture re-appeared, and the child was asked for the “meaning” with *And what does this one like? What does it like?* Therefore, the words in the two conditions had the same amount of exposure and differed only in whether there were retrieval opportunities during the learning period. For each set, learning occurred in two 20-min sessions over 2 consecutive days.

**Figure 1.**
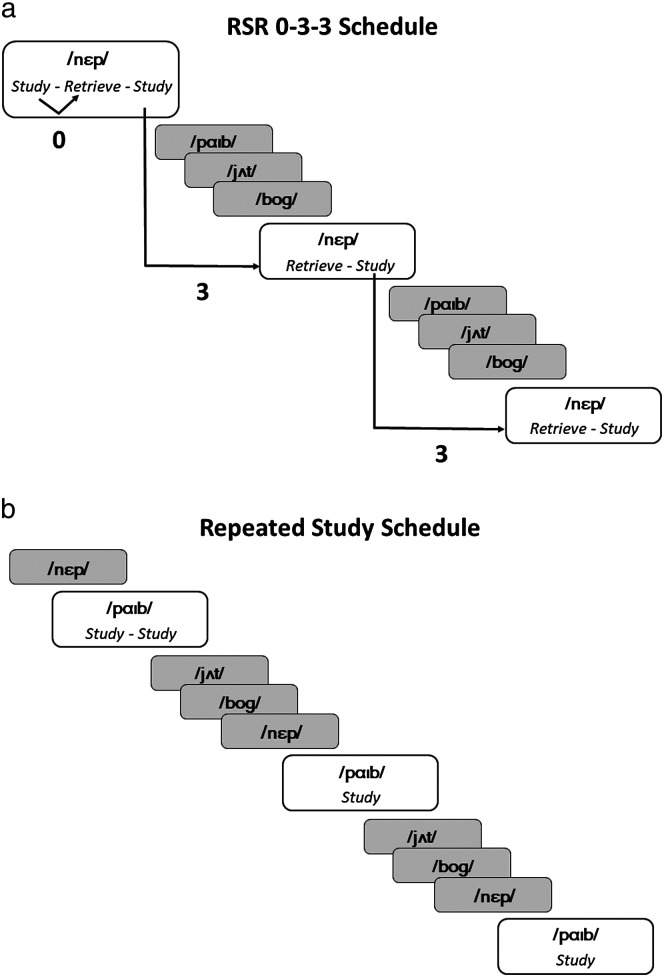
(a) An example of the first block of the learning period in the study of Leonard, Deevy, et al. (2019). In this example, the novel word /nɛp/ is assigned to the repeated spaced retrieval (RSR) condition. In this block, /nɛp/ is retrieved in three instances. Retrieval is immediate in the first retrieval trial. This is designated “0” because there are no words intervening between the retrieval trial and the preceding study trial. For the second and third retrieval trials for /nɛp/, three other words intervened between the retrieval trial and the preceding study trial. For this reason, these retrieval trials are designated “3.” (b) An example of the first block showing a novel word /paɪb/ assigned to the repeated study condition. In this instance, three other words intervened between appearances of each word, but only study trials are employed.

Words in the retrieval condition were presented within an RSR schedule (hereafter referred to as the RSR condition). The first retrieval trial for each word was an IR trial, as retrieval was requested directly after a study trial for the same word. Thereafter, for each retrieval trial, there were three intervening words since the word's previous study trial. A study trial for the same word immediately followed the retrieval trial. For words in the RS condition, the number of study trials matched those used in the RSR condition. Words in the two conditions were interleaved in the presentation list. Figure 1 illustrates the pattern for each condition separately.

After the second session, the children were given a 5-min break and then were tested on the recall of word form and meaning. These tests used the same pictures and requests as the retrieval trials. Following the recall tests, a recognition test was administered in which the children were asked to point to the requested picture from among three alternative pictures appearing on the screen (e.g., *Which one is the /nɛp/? Where*'*s the /nɛp/?*). The children returned 1 week later and received the same tests.

A series of mixed-effects models were estimated, with and without the covariates of maternal education and standard scores on the PPVT-4. The covariates proved to have no bearing on the results. Neither covariate was correlated with the children's novel word recall scores.

**Figure 2.**
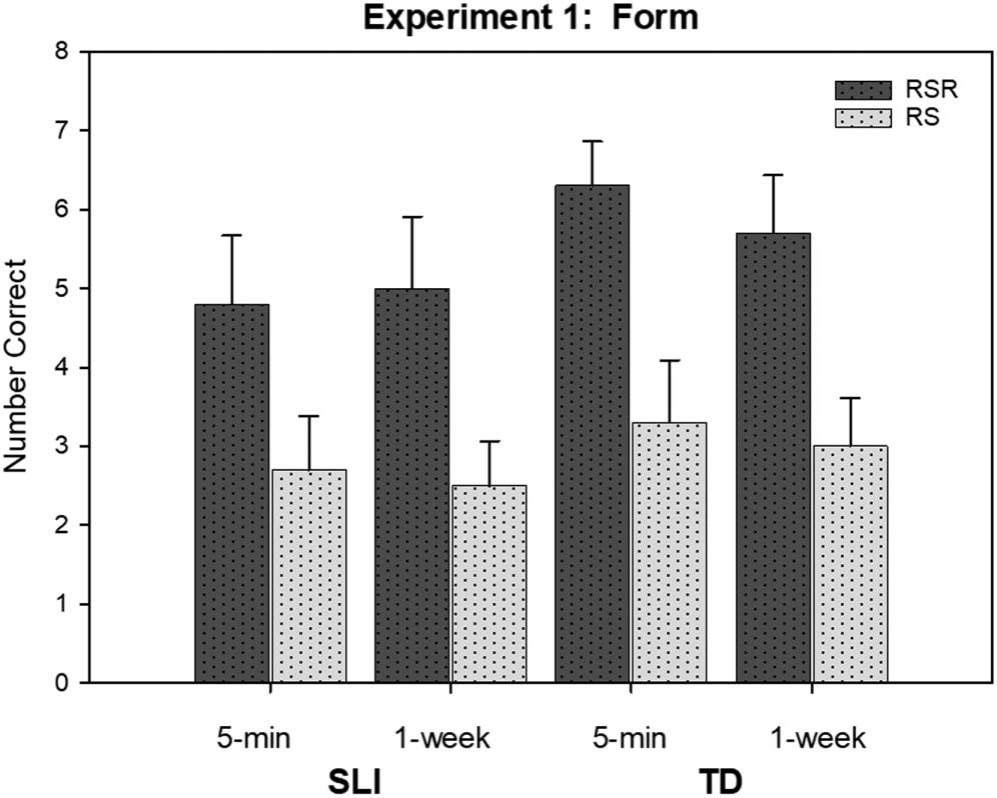
The (unconditional) mean number of items correct on the recall test at 5 min and 1 week for novel nouns in the repeated spaced retrieval (RSR) and repeated study (RS) conditions by children with specific language impairment (SLI) and children with typical language development (TD) in the study of Leonard, Karpicke, et al. (2019). Error bars are standard errors.

Figure 2 provides an illustration of the results for word form. There was a large effect size for learning condition, favoring the RSR condition over the RS condition. This difference applied across groups and time periods. Children's retention did not decline from testing immediately after the learning period to the 1-week point. This stable retention held for both learning conditions and both groups of children. The trend toward higher retention scores for the TD children reflected in Figure 2 was not statistically reliable.

Because we were interested in discovering whether repeated retrieval had potential as a future clinical treatment procedure, we determined how many of the children with SLI showed a difference between the two learning conditions in their recall scores. Nine of the 10 children showed a difference favoring the RSR condition. We note also that this difference was seen in children whose recall was relatively poor as well as those with rather strong recall.

The results for meaning were similar to those for word form. The meanings of the words in the RSR condition were retained better than those in the RS condition. Retention was maintained over 1 week. The two groups of children were very similar in their meaning retention. Both groups' scores were higher for meanings than for word forms. The recognition test proved insensitive due to ceiling effects. Scores were high for both learning conditions, time periods, and participant groups.

The results for word form and meaning recall represented a stringent test of the effects of repeated retrieval. Half of the words presented in a study trial subsequently appeared in a retrieval trial. Therefore, it is possible that, during a study trial of a word in the RS condition, the children anticipated being asked to recall the word soon thereafter. If so, they might have engaged in covert retrieval, which could have had a positive effect on learning. Notably, though, even if this occurred, it was not enough to offset the clear advantage held by the RSR condition.

### Experiment 2: RSR Versus Repeated IR

Our first study showed clear advantages for retrieval over study when words in the two conditions were equivalent in the number of exposures provided. However, even though the RSR condition employed spacing, we could not be certain that spaced retrieval—as opposed to any kind of retrieval—was responsible for the effect. The comparison condition of RS offered no opportunities for retrieval until testing after the learning period.

Accordingly, in our second study, we compared alternative retrieval conditions, one involving RSR and the other involving repeated IR (hereafter referred to simply as the IR condition; Haebig et al., 2019, Experiment 1). For this study, then, the two conditions were equivalent not only in amount of exposure but also in number of retrieval trials.

Again, the two groups (16 in each group) were matched for age (*M* age = 5;0 [SLI] and 5;2 [TD]). The format for the learning sessions and testing resembled those used in the first study, with a few exceptions. Twelve novel words were taught instead of eight. Four of the words were among those used in the first study; the remaining words were all two-syllable words (e.g., /pobɪk/, /meləp/). Because more words were used, we reduced the spacing between study and retrieval trials from three intervening words to two. Testing was the same, except the recognition test was administered only at the 1-week point. Data analysis followed the same procedures as in the first study. Again, applying the covariates had no effect on the results, and these were not correlated with the children's recall scores.

**Figure 3.**
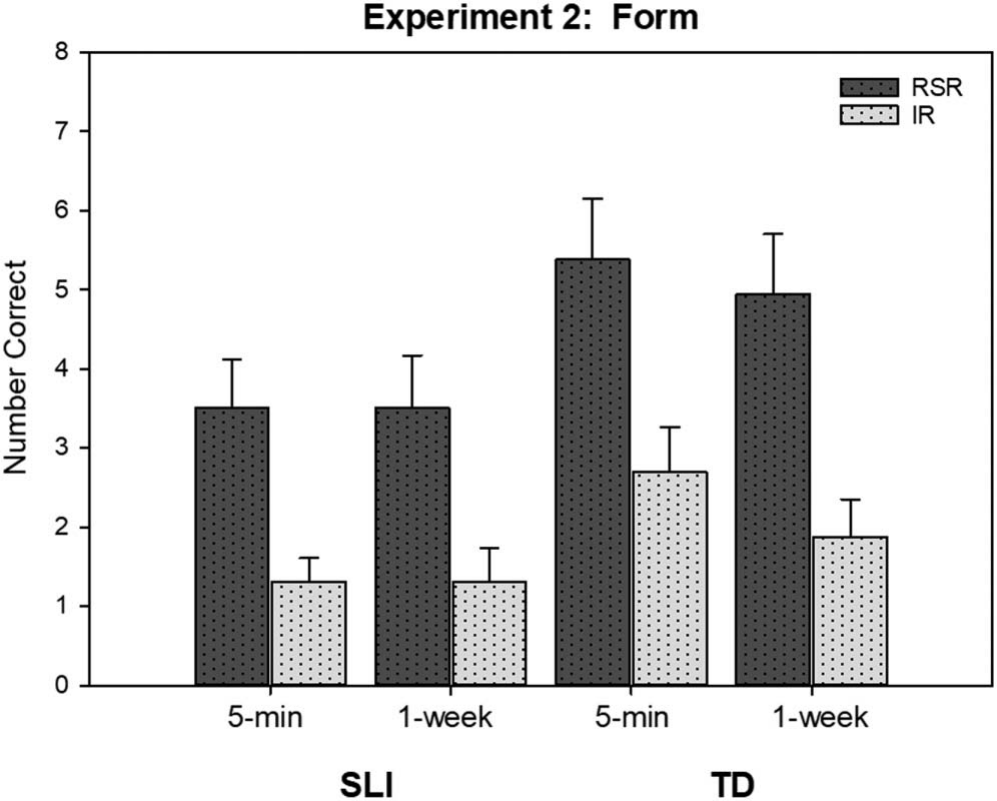
The (unconditional) mean number of items correct on the recall test at 5 min and 1 week for novel nouns in the repeated spaced retrieval (RSR) and immediate retrieval (IR) conditions by children with specific language impairment (SLI) and children with typical language development (TD) in the study of Haebig et al. (2019, Experiment 1). Error bars are standard errors.

The results for word form are illustrated in Figure 3. A large effect was again seen for learning condition; the RSR condition resulted in greater retention than the IR condition. For both conditions and participant groups, words recalled 5 min after the learning period were retained 1 week later. We found a difference favoring the TD children over the children with SLI, but only for the 5-min test. The difference did not hold at the 1-week mark.

Again, we examined the recall scores for the children with SLI and noted that 13 of the 16 children showed better recall for words in the RSR condition. This was true for children with relatively low recall scores as well as those with relatively high recall scores.

The equivalent number of retrieval opportunities in the two conditions ensured that advantages seen for the RSR condition could not be attributed to having had or not had practice in producing the words. In fact, inspection of the children's responses during the learning period reinforced this point. During the learning period, IR trials had a high success rate, owing, presumably, to the immediacy of retrieval following a study trial. This meant that the children actually produced the word during most of the IR trials. In contrast, the early RSR trials for each word often resulted in the children responding with “I don't know.” Correct retrievals gradually increased, but due to the low levels of success early on, the total number of productions of words in the RSR condition was much lower than the number in the IR condition. Clearly, then, given the advantages seen for the RSR condition at the end of the learning period, production practice was not a decisive factor.

The advantages for the RSR condition also held for meaning. Consistent with our other findings, retention was stable over the 1-week period. Unlike our first study, however, the TD children recalled more meanings than the children with SLI. Note that we included more words and meanings in this second study. Recognition testing yielded somewhat similar results; the TD children recognized more words than the children with SLI. The latter group showed an advantage for words in the RSR condition. The TD children performed at ceiling, so we could not determine if a learning condition effect was present.

### Experiment 3: Receptive Measures of Retrieval Benefits

In our first two studies, recognition tests were our only means of assessing learning condition effects in the receptive modality. Unfortunately, ceiling effects for both groups in the first study and for the TD group in the second study hampered our ability to identify possible differences. Based on the episodic context framework we have used as a basis for our studies, the learning that results from RSR should apply as well to receptive measures. The problem is that recognition tasks such as the ones we employed can lead to correct responses even if a child's representations of the target words are phonetically imprecise—possessing only enough detail to allow the targets to be distinguished from the (phonetically quite different) alternatives.

As a more suitable alternative, we turned to electrophysiological measures in our third study (Haebig et al., 2019, Experiment 2). We used a match–mismatch task in which detection of a mismatch between a picture and the immediately following word usually elicits a neural N400 response at particular electrode sites (P3, Pz, P4, PO3, PO4, O1, Oz, O2). Twenty-seven of the 32 children (SLI = 14, TD = 13) in our second study (comparing the RSR and IR conditions) served as participants. The children were tested immediately after they had completed the 1-week recall testing. We hypothesized that the children would show N400 responses with larger amplitudes to mismatches involving the novel words in the RSR condition than to mismatches involving the novel words in the IR condition. Our rationale was that, if words were better learned in the RSR condition, then corresponding pictures would elicit a stronger priming effect for these words. If, instead, a different (incorrect) word was heard, the neural response to this mismatch would be robust and more reliable than mismatch responses to words less well learned.

The results were in line with this expectation. N400 amplitudes were, in fact, larger for mismatches involving words in the RSR condition than for mismatches involving words in the IR condition. Somewhat surprisingly, we found no difference between the two groups of children; both groups showed very clear amplitude differences between learning conditions, and the magnitude of these differences was similar in the two groups.

### Experiment 4: Benefits Beyond the Specific Items Learned

In our first three studies, spaced retrieval effects were seen for novel words representing nouns—names of exotic plants and animals. We used only a single picture for each word. Therefore, it is possible that the benefits of spaced retrieval applied only to the specific word–picture pairs used in the learning period. For studies of memory, this may be sufficient, but for studies (such as ours) purported to study the learning of new words, additional steps are needed. Words usually apply not to one specific referent but to a class of referents. Therefore, we took the next step of determining if retrieval effects hold even when a newly learned word is subsequently tested in the context of an appropriate but wholly new referent.

We pursued this idea by using novel words representing adjectives. Adjectives refer to attributes that can distinguish different exemplars of the same category (e.g., a yellow flower, a red flower, a white flower) or refer to an attribute that is common across different categories (e.g., a yellow flower, a yellow bus, a yellow pencil). Therefore, adjectives represented a useful way to test whether retrieval-based learning resulted in something akin to real word learning, where the word is not limited to the original referent but is applied to new referents sharing the same attribute.

**Figure 4.**
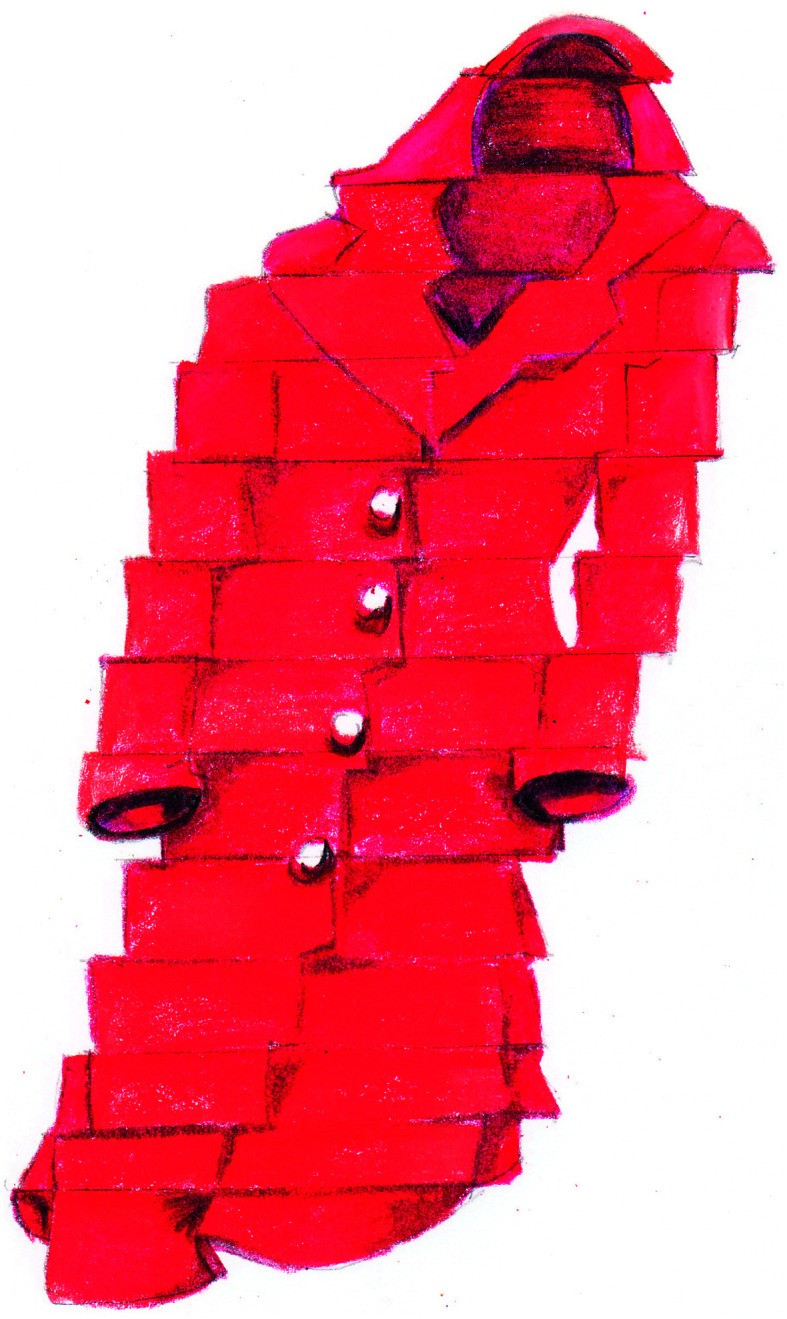
Example of a drawing used to illustrate a novel attribute in the adjective study of Leonard, Deevy, et al. (2019). Copyright © Stephanie Funcheon. Reprinted with permission. This image does not fall under the Creative Commons license.

In this fourth study, we recruited children with SLI (*n* = 14, *M*_age_ = 5;3) and same-age peers with typical language development (*n* = 13, *M*_age_ = 5;3) and taught eight novel words (e.g., /*zogi*/) that referred to an artist's drawings of novel attributes associated with common objects (e.g,. a /*zogi*/ truck, a /*zogi*/ cow; Leonard, Deevy, et al., 2019). An example of a drawing of a novel attribute appears in Figure 4. As in our other studies, a within-participant design was used. For each child, half of the novel adjectives were taught in an RSR condition and half in an RS condition. Testing occurred immediately after the second learning session and 1 week later. During recall testing, we included generalization items—drawings of the attributes associated with objects that were not seen during the learning period (e.g., a /*zogi*/ car, a /*zogi*/ table).

**Figure 5.**
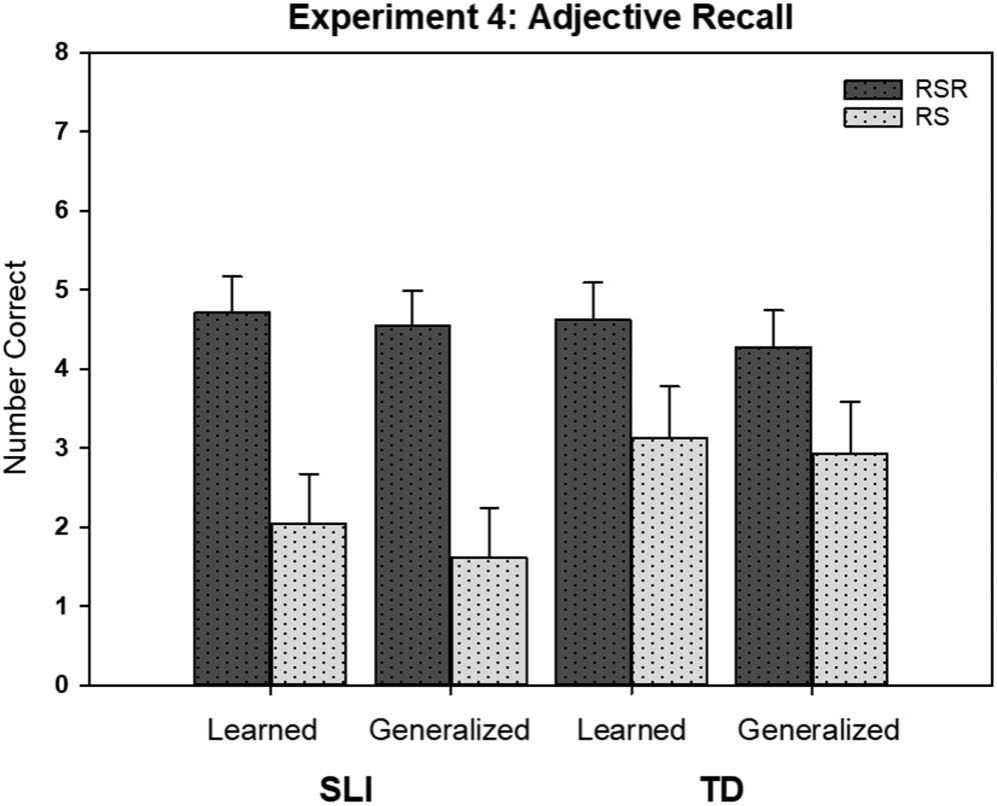
The (unconditional) mean number of novel adjective items correct on the recall test for learned and generalization items in the repeated spaced retrieval (RSR) condition and the repeated study (RS) condition by the children with specific language impairment (SLI) and the children with typical language development (TD) in the study of Leonard, Deevy, et al. (2019). The figure reflects the similar behavior of the learned and generalization items when considered within each learning condition and participant group. Error bars are standard errors.

Mixed-effects models were estimated. For this experiment, we included EVT-2 scores as well as PPVT-4 scores and maternal education as covariates. Only the EVT-2 scores were correlated with children's recall test scores (*p* = .050), but again, the covariates had no influence on the results. Recall was stable, with no differences across the two time points. Figure 5 provides an illustration of the other findings. There was a very large effect for learning condition, with greater recall in the RSR condition. The two groups of children showed comparable recall. Of special importance in this study was the finding that test items representing items not included during the learning period yielded accuracy as high as the items actually used in the learning period. This type of generalization suggests that words benefiting from retrieval practice are more than memorized items; they have sufficient flexibility to be applied to new referents.

We inspected the results for the individual children within the SLI group and found that 13 of the 14 children showed greater recall for words in the RSR condition. Again, this proved true for the children with relatively weak recall, as well as those with better recall.

### Relative Strengths in the Word Learning of Children With SLI

There were not as many differences between the two groups of children as we were expecting. In some instances, there was not even a numerical trend favoring the age-matched children with typical language development. However, we do not claim that the children with SLI recruited for our studies were as capable as TD children in their ability to learn words. For example, our studies were not designed to isolate the encoding process (though see Experiment 1 in Haebig et al., 2019, for suggestive data), and as noted earlier, this process appears to be quite weak in children with SLI. Instead, our focus was on retrieval effects, long-term retention, and generalization. Furthermore, we can imagine that had we included a larger number of words to learn, or reduced the number of study or retrieval trials, more participant group differences might have been seen. Note, however, that even with the design details we employed, we were able to observe very strong effects for learning condition in all of our studies.

We believe that, in two major areas, the absence of an interaction with participant group reflected not an insensitivity in our design but rather a relative strength in the children with SLI. In particular, we could not find a trace of evidence that the children with SLI were less able than their age mates to retain information over time. This was true for both word forms and meanings and for adjectives and nouns. Of course, we defined long-term retention as retention over 1 week. It is certainly possible that retention measures covering longer periods could reveal something different. At least as applied to 1-week retention, our results add to earlier findings reported by McGregor et al. (2017, 2013) for young adults with DLD. Together, these findings suggest that longer term retention is not a major obstacle for individuals with this general type of disorder.

We were also encouraged to see that generalization of novel adjectives to new objects was as strong in the children with SLI as in their peers. Furthermore, the fact that the advantages for words learned in the RSR condition were seen for generalization items as well as learned items suggests that retrieval effects are not limited to specific word–picture pairs already experienced by the children.

However, we should make clear how retrieval and generalization interact. Although in our adjective study recall was more limited in the RS condition, the words that were recalled in that condition generalized to the same extent as the (more numerous) words recalled in the RSR condition. This suggests that, independent of the learning condition, the children were able to detect the attribute being referred to by the novel adjective and apply that attribute to new objects. What spaced retrieval accomplished was to allow the children to more reliably access the words that served as the names of these attributes. Thus, spaced retrieval did not create an ability to generalize but rather enabled the children to recall and retain more words that could reflect generalization. This is no small thing; everyday language use requires speakers to efficiently retrieve known words to refer to new things. Without successful retrieval, communication would be hindered.

### Potential Clinical and Educational Applications

Much more must be learned about retrieval effects before we can confidently apply it to daily clinical and educational practice. Additional retrieval schedules should be evaluated, the optimal number of words taught per session should be determined, and other types of words (e.g., verbs) should be included before we are ready to move from laboratory experiments to less controlled work in more natural settings.

Future research should also consider making deliberate attempts to recruit children with SLI whose vocabulary test scores are especially low. This is not to say that the children we recruited were unrepresentative of the preschool-age SLI population. All of the children were enrolled in a language intervention program or were scheduled to enter such a program. Furthermore, in principle, children meeting our selection criteria could have had much lower vocabulary test scores than they did. As noted earlier, other studies have reported age-appropriate vocabulary test scores in children meeting the same types of selection criteria. It is also true that our TD participants showed above-average scores on these vocabulary tests. Although it is possible that these children were unrepresentative of TD children, an argument can be made that the uniformly higher-than-expected scores of the children in both groups across the studies is a reliable characteristic of the local participant recruitment area. Note that, despite higher vocabulary test scores overall, the expected gap in these scores between SLI and TD groups was consistently seen (with very large effect sizes).

Even if our participants with SLI were representative of many children with this diagnosis, there are surely children meeting the criteria for SLI who score especially poorly on vocabulary tests. To ensure that retrieval practice is likely to be beneficial for these children, special efforts should be made to seek these children out. We can offer one observation from our studies that suggests that low-vocabulary children, too, might be assisted by retrieval practice: We found that, in each experiment, RSR produced greater recall than the comparison condition even in the children with the weakest novel word recall. Put differently, retrieval benefits were not confined to children with relatively good word learning skills.

If replication and expansion of this line of research prove successful, clinical and educational application is easy to imagine. For example, book reading with children could include not only the adult's use of each new word with sufficient frequency but also opportunities for the children to retrieve the word. Spacing of retrieval could also be incorporated by having each word's referent reappear in later pages (e.g., “Oh, here they are again! Do you remember what these things are called? What did we call them?”).

Thus far, the evidence is encouraging. Consider, for example, that our RS condition was based on the common practice of ensuring that children hear new words with high frequency, as an aid to learning. In addition, the practice of having the children repeat new words after the words are introduced is also quite typical (e.g., “This is a *saxophone*. That's a long word. *Saxophone*. Can you say *saxophone?*”). Our IR condition simulated that practice, though in a more controlled manner. Now compare the results for the SLI groups in Figures 2, 3, and 5 for RSR with the results for the TD groups for RS (see Figures 2 and 5) and IR (see Figure 3). These figures suggest that the more ideal procedure of RSR enabled the children with SLI to show greater recall and retention than their peers accomplished with the more “business-as-usual” practices. Of course, we found evidence that both groups benefited from RSR. However, to the extent that RSR can only be provided to, say, children qualifying for clinical services, it is useful to know that this procedure might help these children narrow the word recall gap with their peers who are learning under more typical methods.

## Supplementary Material

10.1044/2020_JSLHR-20-00006SMS1 Presentation Video 2019 ASHA Research Symposium: Laurence B. Leonard, Repeated Retrieval Facilitates Word Learning and Recall in Children With Specific Language Impairment

10.1044/2020_JSLHR-20-00006SMS2 Transcript Transcript: 2019 ASHA Research Symposium: Laurence B. Leonard, Repeated Retrieval Facilitates Word Learning and Recall in Children With Specific Language Impairment
