# Supplementary material for: Retrieval Practice and Word Learning in Children With Specific Language Impairment and Their Typically Developing Peers
Source: J Speech Lang Hear Res. 2020 Oct 15;63(10):3252–62. doi: 10.1044/2020_JSLHR-20-00006 (PMC8084525; doi:10.1044/2020_JSLHR-20-00006)
Supplement: Transcript [file JSLHR-63-3252-s002.pdf]

Repeated Retrieval Facilitates Word Learning and Recall in Children With Specific Language Impairment

Thank you Mabel for that very nice introduction, and thanks especially for your tremendous contributions to the field. And thanks also for mentioning this morning my dad's study. (Everyone laughs) Um… I'm really happy to be here and give you, have this opportunity to talk about our project on word learning and retrieval with children with specific language impairment. And before I get rolling, I'd like to acknowledge our principal collaborators, and also give thanks to the National Institute on Deafness and Other Communication Disorders for their support for this project and, and related work that I'll be talking about today. And I'd like to thank also the general, the whole, the Whole Child Language Lab who do, uh, people who do just a lot a great work that allow people like me to stand up here and take credit for it. And here's the disclosure statement. Other than the fact that we have funding that I just showed you a minute ago.

Well we've already been talking about children with specific language impairment. I'm gonna go through the slide rapidly. But just so that we're calibrated, we are also in the projects that I'll be talking about today, referring to children with a specific deficit in language ability, yet normal hearing, no clear signs of neurological damage or disease. The language deficit cannot be attributed to other weaknesses that these children sometimes have in other areas. Vocabulary deficits I'll be talking about today are very common in these kids, even when standardized vocabulary tests might suggest normal ability. And as we know, this recent… trend towards talking about these children as children with Developmental Language Disorder. What I wanna just mention today is that the children I'll be talking about today have met the classic criteria of, of specific language impairment, especially with regard to IQ's within normal limits.

So the vocabulary skills of children, I probably should of added a lot of citation since the people who did this work are out here in the audience today. Many of the mentors, people on the panel, and in the other audience. But we know that these kids have less breadth and depth in their vocabulary than those of their same age peers. And we also know across time that these kids fall further behind their peers in vocabulary ability from preschool through adolescence. For those individuals who are participating, those kids who are participating in novel word learning studies, we know that it requires more exposures to those words to reach the same criterion level that we see in their typically developing peers. And in those novel word studies, we see weaknesses in novel words referring to nouns, referring to verbs, and referring to adjectives as well.

And so what I'll be talking about today is word learning in these kids, and specifically I'll be askin' the question can retrieval practice significantly facilitate these children's word learning and retention. Well of course this question has been around for a very long time in the psychology literature. The best way to remember things in particular is retrieval, contributes something other than just being a neutral assessment of what we have just

learned. You can go back in the psychology literature to the late 1800's and see scholars making some informal observations about that. But the first systematic study that tends to appear in the literature, as far as I could determine, is a study by Abbott in 1909; it was a real systematic study. And I wanted to go and read that, just for some historical background. And when I did, I really found that that psychology article could be traced back to a Master's thesis from the University of Illinois by Edwina Eunice Abbott, a very nice systematic study, the first systematic one that I can determine. I don't know how well you can read it. It's just a scan of the cover page of her thesis. I retyped it, just in case you have, (Several people laugh) you have eyes like mine. Doesn't have that yellowing page look. In the retyping. But it's a really nice study, and Edwina Eunice Abbott was ahead of her time.

Some of the concepts dating back then still ap-- apply now. But I, so that we're all sort of on board with some of the basic notions that will be used in the experiments that I talk about today, I'm gonna talk about a couple a these basic concepts. One being the distinction between repeated study and repeated testing, or repeated retrieval. I'll be using repeated retrieval often as a, just a synonym for repeated testing.

Okay, so maybe to introduce the concept, I'll try to do this quickly, is, let's assume we're doing a little thought experiment. And those folks in this side of the room, you will enter this experimental room, and you'll each have a laptop in front of you. And you're gonna read in that laptop a lot a text that includes science concepts. And you're gonna be studying these science concepts in 5 minute chunks. You're gonna study for 5 minutes, then you're gonna stand up, you know get some water, sit back down, study for another 5 minutes, stand up, get some water, or sit down, whatever, you know. Anyway, you're gonna do this in 5 minute blocks, okay. And at the end of let's say the 6th 5 minute study period, you're gonna take another quick break, and then we're gonna give you a test on your recall of those science concepts. And then you're gonna leave and you're gonna come back one week later, and we're gonna test you again. Now the folks on this side of the room, you're gonna be doing something much the same. You're gonna be studying this material, these science concepts on your laptop, in 5 minute chunks. But for you, after each 5 minute study period, you're gonna slide the laptop to one side, grab a piece a paper, and try to write down everything that you just studied for that last 5 minutes. When you can't remember anything more, then you put that piece a paper aside, you pull the laptop back, and you study for another 5 minutes, okay. Then you're gonna slide the laptop away and get a fresh piece a paper, not looking at the first piece a paper and try to write down again, all the concepts that you can remember. And you're gonna continue to do that for 6 5 minute chunks also. Then take a final test, then come back a week later and take the test again, mkay. It's a very classic paradigm, there are variations in the material and number of trials and so on, but that's the basic idea. In fact, there's a recent meta-analysis that identified 159 studies in the psychology literature that more or less had that kind of theme. And the vast majority showing the same kind a results, which I'll outline here.

So here's an example of one such study. It's one by our colleague who's on our grant, Jeff Karpicke in psychology, and his colleague Roediger. So, first I'm gonna show you

folks on the left how you did. (Laugh) You did really quite well when we tested you after you finished that 6th 5 minute block. But when you came back a week later, you didn't remember quite so much. Now you folks over here did quite well also when we tested you after that 6th 5 minute block. But when you came back a week later, you did considerably better than the folks on this side a the room, mkay. It's a benefit really of, of retrieving information. So it's not just a, a neutral event, it actually sees to bolster retention. So that's one basic concept of repeated retrieval versus repeated study.

The second concept that we'll be making use of in the experiments I talk about today has to do with spaced retrieval. And I know some of you are familiar with that concept of spaced retrieval, 'cause you could see it some, in on some of the literature with for instance patients with… with Parkinson's, pacen—patients with Alzheimer's, where spaced retrieval is used as a method to help these individuals remember very life important skills like when to take medication and so on. This notion of spaced retrieval has been in psychology literature for quite a long time. Not really used so much with regard to word learning however.

So, to talk about that, let's go back to another experiment. You folks over here, thank you for agreeing to participate in the second experiment, after going through that first and being soundly beaten by the people on that side a the room. (Several people laugh) Now your job in this study though, is you're gonna be facing a laptop again, and you press a key, and what you'll be doing is you'll be learning the English word translations for Italian words, mkay. So, so when you press the key, you're gonna see an Italian word, fiume, in this case, and it means river, and you're gonna study that for a minute. And then you're gonna hit another key, and you're gonna be prompted that, you'll, you'll get the Italian word, and now you have to provide the English translation. Mkay. So you're testing yourself this time. You have a; this is retrieval, but this is immediate retrieval, right? So then the next word comes along, you learn this pair, you study it, and then you prompt it, and you gotta come up with that English translation for that word, and so on. And it continues on like this for another word, and you're tested again. And then pretty soon we come back to the first word pair, mkay, and you're prompted, mkay. So, just to show you what we just went through here, you've gone through this period of, of seeing; studying it, and then having an immediate retrieval trial, and studying, having another retrieval trial, and so on, mkay. Now, for the folks—oh, and let me just outline here in green, the, just the repetition. Now we're coming back to that first word pair, mkay. Now, for the folks over here, they're also gonna be doing the same thing, mkay. But, when we come to a repetition including one a those initial words again, you're gonna get an immediate retrieval trial. Fiume, okay, you know, what's it mean in English? So you're, um… you're getting now what is often called a spaced retrieval trial. Spaced because we have words intervening between the last time you studied the word, in this case river, the answer, and the time you're asked to retrieve it. So it's, it's referred to spaced retrieval, 'cause we had these other words interspersed, okay. So that's basically the essence of immediate retrieval, which is exclusively what you folks over here had, and you folks who started with one immediate retrieval trial, but then went into a spaced retrieval condition, mkay.

So when you look at results for this kind of study, you'll see, that, uh, I'm sorry, you know, now I'm showing the, the immediate retrieval versus the spaced retrieval, that, you know, so you can kinda see what I was just talking about visually. When you look at the results of this kind of study, again I'm gonna be referring to one of the, the Karpicke studies. For immediate retrieval, your first recall is gonna be you know very accurate, but 2 days later, there'll be a lot that you don't remember. When you try the spaced retrieval, here's where I'm referring to the first spaced retrieval trial. You're not gonna be at 100%. For, for one thing, you're probably surprised that you're being asked without hearing the answer at, at that point. So you're not gonna do quite so well initially. But 2 days later, you're gonna do significantly better than the first group, mkay. So, this is an instance of spaced retrieval having an advantage of immediate retrieval.

So I'll be making use of those two concepts then, repeated retrieval versus repeated study, and spaced retrieval versus immediate retrieval.

Okay, we're not the first to apply some of these notions. Not, uh, we might be the first to apply some of them, but certainly not the first for… for the general idea. But thanks to NIDCD, we've been able to look at this question rather comprehensively, which is, got some advantages. First, some possible practical implications down the line, is what we often hear about the importance of hearing a lot of words, for children's vocabulary development. But how 'bout even, regardless of what the input is, if you hold that input constant and you give children the opportunity to retrieve those words, might they learn and retain those words more successfully? And because we're able to look at this issue of retrieval from different angles, we're hoping that we can get a better understanding of some of the mechanisms that might be intact or impaired in these kids.

Okay, so the first experiment really is the repeated study versus repeated retrieval kind of condition. For this study, we had 10 kids in each group, they average age 5. The SPELT-P2 is one of the main selection criteria, so there's no surprise that the kids are gonna differ there. Now we did not use vocabulary test as a selection criterion, we used the vocabulary test simply as additional descriptive information when we described the kids. And this is something that you'll be seeing again in my talk. You know, for a couple of experiments, we often see significant differences 'course between the groups favoring the typically developing kids. But SLI kids who tend to fall in the average range or low average range, but definitely within the standard deviation of the mean, if not considerably higher, in our work.

I'm gonna go through the procedure in some detail, but bear with me, even if I get, you know, bogged down a little bit, because we'll be using the same method in some of the subsequent experiments that I talk about. So I'll be able to kinda jump over and get to the results more quickly when I talk about these other experiments. So we had 8 novel words, in this case nepp and pibe were a couple of them. We taught them in two sets of 4. Now importantly, this is within subjects design. So as the kids are learning each set, there'll be two words from the repeated spaced retrieval condition, RSR is repeated space retrieval, and two words taught in a repeated study condition. That same was gonna be true in the second set that gets learned subsequently. Mkay. These words in the two

conditions are matched for the number of times the children heard the word. So we have to, you know, control for exposure. And they were essentially novel nouns referring to exotic plants and animals that Karla McGregor kindly sent our way; the pictures of the plants and animals that is. So, it's just an example of a study trial. All of our conditions had study trials. So a child would see this on a the laptop and hear, "This is a nepp, it's a nepp, a nepp likes birds." For an example of another one; "This is a pibe, it's a pibe; a pibe likes butterflies." Now for words in the retrieval condition, there were also retrieval trials. So for word form, by word form I mean asking for the actual word nepp in this case, the child would hear "What do we call this? What's this called?" Or to ask for the meaning. By meaning, I'm referring to what it likes. "And what does this one like, what does it like?" And so you know, just another example, here's what's this called. What do we call this?" "It's a pibe." "What does this one like?" What does it like; butterflies." And so on. Okay.

Again bear with me, but once we get through this, I won't have to repeat myself and it will go more smoothly.

So this is an example of one set. In this particular set, the very first novel word being presented is the novel word nepp, in red. So the study trial refers to again, the picture comes on, the kid hears "This is a nepp, it's a nepp, a nepp likes birds." Picture goes off, comes back on, and the kid gets a retrieval trial; "What do we call this? What's this called?" Okay, notice that's an immediate retrieval event, right? and we're gonna call that a zero trial, simply because there are no intervening words between the last time the child hear nepp and the time the child is asked to retrieve nepp, right? So the kid tries to, you know, come up with the word nepp, and what it likes. And then we move on just to another study trial in this instance. So the kid hears again, "This is a nepp, it's a nepp, a nepp likes birds." And then we move on to the next words, pibe, yutt, and boge, and then eventually we get back to nepp again. But notice when we get back to nepp again, we start with the retrieval trial. So the picture of the nepp shows up and the kid hears "What is, what's, uh, what do we call this; what's this called?" So the kid did not have an immediately prior study trial. So you can see that's an example of spaced retrieval. We call that a 3 trial; why; because there are 3 intervening words, pibe, yutt, boge, from the last time the child heard the word, nepp in this instance, and the time the child is asked to retrieve it, 'kay. And so it goes. Another study trial, then more intervening words, and we come back and nepp, nepp appears again, but right off it's a retrieval trial, the kid has to come up with the word, so that too is a 3 trial, because there are these 3 intervening words, mkay, so that's the general idea. Okay.

Now we also had of course, as within subjects design, words in the repeated study condition within the same set. So in this instance, pibe would a been one of the study words. So the child sees the picture, this is a pibe; it's pibe, a pibe likes butterflies. Picture goes off, comes back on, here's another study trial, it's a pibe; pibe likes butterflies, and so on, mkay. So otherwise it's, it, it follows that, that basic pattern. So two retrieval words, two study words per set. Mkay.

Then what happened is we, we conducted this across two days, just two sessions, across two consecutive days, a total learning period of 40 minutes, mkay. and then we gave the kid a 5 minute break, and then we tested the child. The testing involved, this is the 5 minutes after the last set, uh… uh, period in the day 2. The testing involved a word form test, which is like our retrieval trial; you know what's this called, what do we call this; nepp. And what does this one like, which is our word meaning test; you know likes birds or butterflies and so on. We also had a recognition test, which was just simply a picture pointing multiple choice. In this case we only had 3 alternatives. We change that in a later experiment, but you know, which one is the nepp, where's the nepp, the child just needed to point to the picture on the laptop.

Okay. We had, um, we… oh, let me continue and say that that was 5 minutes after this, the learning period was done. But then the kids came back one week later and got the same tests again. So we did testing of longer term retention as well. Okay. So we, um… ran a series of mixed effects models. We used the covariance of Peabody picture vocabulary test, and maternal education, and we looked at models with and without the covariance. It turned out the covariance had no bearing in the results. I'll be presenting values that happen to have the covariance in there, but it just, it didn't affect anything.

So I'd like to pay attention to a few things. This is What's This Called; nepp, pibe, and so on. First what I'd like you to do is to compare the dark bars with the lighter gray bars, and you'll see across the board, they repeat; the words learned in the repeated space, retrieval condition were recalled better than those… learned in the repeated study condition. It's a very, very strong effect. Next, what I would like you to do is to compare the 5 minute to the one week mark, and you'll see that if the children remembered it at the 5 minute point, they tended to remember it one week later. We're actually quite pleased about that result.

Now it looks as if there's a group difference where the typically developing kids having better recall, it turned out not to be significant. And we had our stats person run some analyses. It turns out that if that trend were to continue, the trend you see in front of you, we would, to reach significance, we would of needed to have 65 children in each group. And oh, so we are absolutely not com—we are not, we are not claiming that these kids were equivalent; that our SLI kids were, got as good as the typical kids; not at all. We don't believe that at all. But what we are saying is, given the design, the number of words we had, the number of exposures we chose, we were plenty sensitive to get condition differences, but not sensitive enough to pick up the typically developing versus, um, SLI and difference. But from a practical standpoint, you wouldn't wanna use this design and then run 65 kids in each group, just to, to show a difference. Some things might wanna be modified in order to show a, to get a more sensitive distinction between groups. Well that had to do with word form, things like nepp and pibe. How 'bout, you know, "What does it like;" birds, butterflies, and so on. Again, if you compare the dark bars with the light gray bars, there wasn't; uh, there was a significant effect. It wasn't as large as for a word form. These kids were; all the of the kids, were simply better at remembering these arbitrary associations between birds and a nepp, and butterflies and a pipe, and these other ones. We made sure that the pictures showed no, provided indi—

indication as to whether birds or butterflies or, some of the other things, rain and snow, would be involved at all. So these were arbitrary associations. But after all, these were real words these kids knew; birds, butterflies, and so on. And what they were able to do is, after enough trials, associate them with the pictures. But nevertheless, there is an effect for a condition, again repeated space retrieval having the edge over repeated study.

Now again, if you compare then the 5 minute and the one week period, you see really good stability. If learned at 5 minutes, they held on to it one week later. This time we clearly didn't get any group differences, but a lot a that had to do with the kids who were bumping up against ceiling levels I think.

Okay, and recognition; recognition was kind of a bust because everybody did rel—really well with those 3 alternatives. It didn't take much; it's kind of a shallow representation of a word, to pick it; to recognize it and pick it, distinguish it among other alternatives that are right there on the screen. So just wasn't a very sensitive measure at all.

So what do we have so far? Well for form recall, nepp, pibe, and so on, we found repeated spaced retrieval having the advantage over repeated study. Same thing for meaning, birds and butterflies. The one week scores were as good as the 5 minute scores, and our recognition test was just showing ceiling, ceiling effects, nothing distinctive.

So we move to experiment two. Uh, repeated spaced retrieval versus repeated immediate retrieval. And our logic here is that, well what were we doing in experiment one; in experiment one we were comparing re—yes, it was repeated spaced retrieval, but it was being compared to repeated study. There's no retrieval involved in that second condition. So was it spaced retrieval or just retrieval of any type? That's what we're really asking here. So in this experiment two, what we did was we had two conditions, each involving retrieval. One involving spaced retrieval. I'm gonna call that a 0-2-2, is a lot like that 0-3 thing I—3-3 I showed you. The reason why we made the spacing a little narrower is 'cause we added the number of words we were asked to; well we needed to add more words, and we also added some two syllable words. Most of the words were two syllable words which are harder and, and so we went to an 0-2-2 schedule. But we compared that to a no spacing schedule of immediate retrieval. Okay. so, everything was a 0-0-0, nothing in between the last time the child heard the word and the time the child was asked to retrieve the word. We had again 5 year old's. This time we had 16 kids in each group. SPELT-P2, very clear differences. But once again in the Peabody, group differences yes, but SLI kids following in at the normal range according to the manual of the test. I should always say that. So our kids do tend to test a little high in our, in our region.

The method was exactly the same in terms of the kind of procedure, the kind of testing. The only difference, and the reason I'm showing you, this particular slide is for the recognition test, that before had 3 alternatives on the, on the screen, now we have 4, just to try to make it a little difficult. And we administered only at the one week point rather than the 5 minutes and the one week point. So, same mixed effects models, covariance. Did not have a bearing on the results. So again, if we could compare the dark bars with

the light gray bars, you'll see again, a very strong effect. This is where kids are having to remember words like the nepp, nepp and pibe, and (sounds like pobic and kudip) and some others that were favorites of ours at least. Very clear effects for a learning condition. This time though, it's repeated spaced retrieval versus immediate retrieval. So we're not only controlling for amount of exposure across the two kinds of words, but also for the number of retrieval opportunities for the two kinds of words, mkay. You can see by comparing 5 minutes to one week, a lot a stability. If they could remember it in 5 minutes, they tended to remember it at one week. And this time we did get a group effect, however it turns out it was just restricted to the 5 minute test. So for the, at the 5 minute test, the typically developing kids were retaining more than the, the SLI group. Now it turns out that was no longer significant at one week, but that could have been kind of a, a fluke, a sampling phenomenon. What happened, as you can see, is the typically developing kids slipped just a little bit over that one week period, whereas the SLI kids, just held, held firm. And, and so, to the point where it kind of slipped out of significance if you will.

How 'bout for meaning? You know, again birds and butterflies and the like. Here again we got an effect favoring repeated spaced retrieval. Again the effect was smaller for meaning than it was for word form, but consistent and clear. Again, good stability from 5 minutes to one week. And this time we got a main effect for group. The typically developing kids had better retention overall than the children with, with SLI. Mkay.

And in the recognition test, you remember we administered it only at the one week point for this study. Again we got a group difference favoring the typically developing kids. The typically developing kids were at ceiling, and it wasn't very sensitive for, for them, but they scored very high, and as a group, better than the kids with SLI. For the SLI kids, we did get a condition effect. They were not at ceiling; they did better on, in recognizing words taught in the repeated spaced retrieval condition, than words taught in the repeated study condition. Mkay.

So where are we then as we do our cumulative total here with this summary page? Again, repeated spaced retrieval had an edge, this time against immediate retrieval. Typical developing kids were strong for form; nepp, pibe, and so on, but only at 5 minutes as it turns out. For meaning, again repeated spaced retrieval had the advantage. The typically developing kids were, um… stronger than the kids with SLI's as a main effect. Again, the one week scores were as good as the 5 minute scores across the board. For recognition, repeated spaced retrieval was better. It applied only to the SLI kids though. The typical kids were at ceiling and better than the kids with SLI as a main effect.

Well, so what I pointed out then, and I've tried to play up a lot a the good stuff, but our recognition test, for Experiment One, both groups were at ceiling. Experiment Two, we tried to make it a little bit tougher. But even then, the typically developing kids were still at, still at ceiling. They did better than the SLI group. But we were asking, you know, we really want to find maybe a better receptive measure than a simple picture point and task. Because, whatever thr—our theoretical framework, which I'll touch on a little bit

later on, is certainly one where retrieval effect is, is not just a production phenomenon that we're trying to teach, it's not an expressive language ability that we're teaching in retrieval. In fact, think about that last experiment I just presented. The kids had the same number of retrieval opportunities. In fact, if you're to look at the amount of productions that the kids had, you can bet that the kids in immediate retrieval condition, had plenty of production practice. And yet, they didn't retain the words as well is in repeated spaced retrieval. So it's not just an expressive language phenomenon, it's, it's something deeper than that. And we wanted to tap into that in a way that the recognition tasks didn't do so well. So, we thought about some electrophysiological measures, specifically the N400 in a picture match/mismatch task, and thankfully we had in our grant, Chris Webber who's an ERP expert, and she worked with our postdoc at the time, Eileen Haebig who's now an assistant professor at, at LSU, to develop a, a match/mismatch N400 task. But first we needed to test the feasibility of this, because you know, this is an age group in a group of kids where you know, we're not absolutely sure what we're gonna get, even with a more conventional task. So, so Chris Webber and, and Eileen Haebig started off with a, a… a real word task, one that's been in the literature for quite some time. Some of you might of run this exact task. So it's one where we have a very common picture with a common name is presented, and the child would hear its name. So a dog would be presented, and the kid would hear "Dog." But in the mismatch condition, the same kind of familiar picture would be shown, but the kid would hear the mismatch "Boat," even though it's a picture of a dog. And when there's this mismatch, and it seems to be on a, un, for the semantic domain, what you classically get is what's referred to as an N400. This is sort of an idealized, nice spiky looking N400, but that's what you would get in comparing the match versus the, the mismatch in this particular case. Again, it's kind of in a semantic domain.

Well, we ran this task on our kids in Experiment Two. The repeated spaced retrieval versus immediate retrieval. We, we ran them on this feasibility task with real words, and we found very clear effects where the mismatches were really eliciting a very clear N400 for both groups, both the SLI kids and the typically developing kids. And this gave us confidence that we can kinda move on to apply this knowledge, this paradigm basically, to the kind a questions we really wanted to ask. So then, we called back our, our Experiment Two kids a couple days after they'd finished that final one week testing, and… we… basically asked the following: Okay, are N400s more prominent for mismatches involving novel words that are better learned? That's the usual assumption, right? I mean that if a word was really solidly learned, really solidly known, then when there's a mismatch, that, that should be detected pretty readily, and it might come in the form of a very, you know, the larger amplitude N400 for that mismatch.

So applying that concept to the experiment I talked about in Experiment Two, are the match—the match/mismatch amplitude differences greater for words that were in the repeated spaced retrieval condition, than for words that were taught in the immediate retrieval condition, okay? It's kind of a stringent test really, because the kids didn't remember every word in the repeated spaced retrieval condition, and they didn't remember every word in the immediate retrieval condition. So it was a little challenging to say the least. So applied then to our novel words, this would be a case where the kid

would see the picture of the nepp and hear it; that's a match. The kid would see the picture of the nepp but hear pibe, that's a mismatch. Okay. Chris Webber and, and Eileen identified, based on the, the established literature of the regions of interest. I'm gonna be showin' you the results for the, the SLI kids. I think in the slide they're called DLD, but the, they are our Experiment Two kids that you've just heard about already. And I'm gonna show you first this, in fact, only the slides for the SLI kids. The slides for the typically developing kids looked very much the same. There's no group difference.

So what I wanna do is I wanna identify first in the upper left, you wanna look at the immediate retrieval condition. IR is the immediate retrieval condition. You can see the match and the mismatch wave forms. The red and the black are just laying right on top of each other. It's as if the kid is not really detecting a semantic mismatch, in again the case of mismatches. In contrast though, if you look at the lower left, these are the words taught in the repeated spaced retrieval condition, and you can see there is some separation, mismatch from the match. Now you don't get a real clear spike N400, in fact, what we get is this separation that actually goes on for a longer duration than, than we would of expected. Although we didn't know. Where in the first, we're, you know, we're just trying it out with this kind of paradigm. But the TD kids, the typically developing kids looked in the same way. It wasn't just what spike for them in this kind a long looping separation for the SLI kids, the TK kids looked at it in the same way.

And, a, a different view can be seen if we look at the following. If we're to sep—if we were to subtract the amplitudes for the match trials from the amplitude for the mismatch trials, what you see in the lower left for the immediate retrieval condition are means right around zero, which really matches what we saw in the preceding slide with the, the wave forms, and the red and black were pretty much right on top of each other. But on the other hand, if you look in the upper right for the words taught in the repeated spaced retrieval condition, you'll see that sure enough, when you subtract the match, the amplitudes for the matches from the, the amplitudes for the mismatches, you get a clear separation. No group difference, both are showing very clear effects for the mismatches when taught in the repeated spaced retrieval trial. So we were quite pleased about that result, because now we're talkin' about a receptive task.

So as a measure of receptive processing, ERP showed a similar condition effect for both groups. A larger N400 response to mismatch, and the spaced retrieval condition showed, this is our interpretation, showed a stronger expectation of the correct word, for words in the repeated spaced retrieval, and therefore better learning, and then so where there's a mismatch you get a greater amplitude when there was that mismatch.

Okay. So just adding that in red here, fur a running summary here. So let's go to Experiment Four. Dealing with adjectives in this particular case. It's our most recent study that we've already completed. We have others that are almost completed. Okay, fine, we're moving to another word class, adjectives, which is important to do. But one of the nice things about adjectives, is that of course by definition, an adjective refers to an attribute that can apply across object categories, right? Everything we've been showing before is this little critter is called a nepp; this funny lookin' plant is called a pibe, and

that's kind of it. One could argue that this is really great retrieval, effects are really strong for this item specific recall. But this is more in the case of the adjective study as you'll see I think. We are in fact talking about teaching an adjective that can apply to more than one object category in, in this case. Okay.

14 kids in one group, 13 in another; 5 year olds. SPELT-P2 differences of course very clear. Again, that's Peabody effect of group differences that are very clear, but age level performances by the SLI kids in the Peabody, based on certainly the, the manual at least. Mkay.

Now what we did in this case, since we're teaching adjectives, is, for each novel word this time, we taught two exemplars. So in the upper left for the, the novel adjective taimik, the kids learned these two in the upper left. But the ones in the upper right were held back. We did not present the ones in the upper right. They were presented only at the end during testing, to, to see if these kids could generalize, not only the attribute, but the names of the attribute. Similarly in the lower left, zogi, those two exemplars in the lower left were learned, but the ones in the lower right were held back and used for final testing. Now in order to kinda get the kids already in a mental set where we're not just doing this kind a rote learning, we had a familiarization phase first, which is very, very brief. If you look again at the upper left, the first thing the kids would see is they would, the child would see two, the, the pictures of these two objects together in the upper left, and would hear something like "Look; these are very taimik." And the next item we presented, which is exactly the two pictures in the lower left, and the kid would see those two pictures, and here "Look, these are very zogi." And so on. So there's this familiarization period to kinda get the kid off the time to thinking that this truck thing had this name. It was, it was perhaps this attribute that had the name. But beyond that, we went on, using the same kind a procedures we've already been talking about. It was a, within subject design. This time we compared repeated spaced retrieval and repeated study. For the study trials, and all conditions had the study trials, this was just another attribute, one of my favorite s, that's why I'm showing it to you. and the kid would see this and hear "This coat is naffy, it's very naffy. This coat is really naffy." For words in the… Oh, and then if, for words in the retrieval condition, the retrieval trial would go something like this: The kid would see this picture and, and here, "Tell me about the coat." "The coat is very…" then we have to provide the, the novel adjective name.

Mixed effects models. Covariance used. Covats—covariance had no, no bearing on the results. So let's look first of all at the dark bars versus the light gray bars, and again very, very strong effect favoring the words in the repeated space retrieval condition. This time we're goin' back to a comparison to re—repeated study however. Mkay. We're changing so many things and moving the adjectives that we wanted to keep something a little bit constant too from, from an earlier thing. Clear condition effects, compare time 5 minutes to one week, and very stable learning if they learned it in 5 minutes, they tended to learn, learn it at one week. I might of said that on here 5 weeks. Oh, at, 5 minutes in one week. Five weeks would be nice. (Laugh) We'd still be on the study. 5 minutes versus one week, very stable retention. This time we did not get a group effect. There was really no clear signs that, whatsoever going on here.

I wanted to show you another slide that looks similar, but this time I've broken down learned versus generalization items, mkay. and… the rest really looks the same. But what I wanted to point out is that the difference between repeated spaced retrieval and repeated study that we see in the learned items, these are the ones who went through the learning period, it holds also for the generalization items that these kids didn't see until testing at the end, okay. so we were really pleased about that, mkay.

One thing else too, is that way, by way of preview of something I'm gonna be saying towards the end is, I do want you to look though at the light gray bars for generalization, and, versus learned. And what you'll see, that even though the words taught in repeated study, were not retained as well as the words taught in repeated space retrieval. Nevertheless, if the kid learned it at 5 minutes, the kid tended to hold onto it at one week. It's gonna be relevant actually in some of the comments I made. So retrieval wasn't great for, in that condition. Testing wasn't great in that condition. But what they did retain, they could kinda generalize. It would be relevant later on. Mkay.

So that was very encouraging. Now we also had in, at the one week point, an adjective recognition. It applied for generalization and learning. I'll just show you the overall one here. What we found is the… for the SLI kids, we got a condition effect, where words were recognized better if they were in the repeated spaced retrieval condition, than in the repeated study condition. That applied to the kids with SLI. For the typically developing kids, they were higher in general. There's no condition differences. The typically developing kids were significantly better in their recognition, but only for the words in the repeated study condition. For the words in the repeated spaced retrieval condition, the two groups were comparable in their recognition. Mkay.

So if we just add the results in red now for Experiment Four on adjectives to the rest, again repeated spaced retrieval had an edge. It, again was over a repeated study. Retention of the one week was just solid. For recognition, repeated spaced retrieval had an edge. It was only the SLI kids who showed it. The typically kids, typical kids were higher, and the typical kids were better than the SLI kids, but only in the repeated study condition. For the repeated spaced retrieval condition, the two groups are very similar. And now we could add generalization to Experiment Four, since we've added adjectives, and repeated spaced retrieval tended to also be better, clearly was better than repeated study. But now these are generalization words that went over and beyond the exemplars that were used during the learning period.

So, interpretations. The kids with SLI were just as responsive as the typical kids to the re, repeated spaced retrieval condition. The kids with SLI were similar to the typical kids in retaining information from 5 minutes to one week. I think that's a, a, an important finding. The children with SLI were similar to the typically developing kids in generalizing words who knew objects with the same attribute.

So some additional thoughts here. First, one thing I wanted to say is that we think we were performing a pretty stringent test in this whole retrieval idea. Why? Because it was

a within subjects design. So within each set, these kids would get a word that was taught in repeated spaced retrieval. The very next word might be a repeated study word. So if the child sees nepp, and this is a nepp, this nepp likes birds. Then the picture comes back on; "What's this called?" Then the picture of the pibe comes up, and the kid sees "This is a pibe, it's a pibe, a pibe likes butterflies." Picture goes off, picture comes back on, but the kid is not required to retrieve it in this particular instance. But there could well have been an anticipation by the child that retrieval was expected, at least in the early phase of this study. Eventually the kid might a caught on. But, so there could a been some kind a covert rehearsal, covert retrieval, we don't know, it was covert. But if there was, it wasn't enough to boost the kid's repeated study performance up to the level of repeated spaced retrieval. So in that sense, we think it was a pretty fair and stringent test.

This is something that goes back to something I said before. I wanted to make kinda clear what we think is going on with the advantages of repeated spaced retrieval, okay. It's, uh, maybe I shouldn't of put so many words here, because I wanted to sort of gesture and, and, uh, say stuff out loud. But, so what we think is going on is we think the kids with SLI were, were capable of first identifying, detecting the attribute that was being shown, and we think based on the generalization results, even for repeated study, that these kids could detect and recognize the attribute and apply it to different visual images that had those perceptual features, including the generalization items that were held back until the end. But what we think the advantage of repeated retrieval is, is that repeated spaced retrieval allowed the kids to retrieve more reliably the words that applied to those attributes that they could recognize. So I, I, so it's great that they can detect it, and great that they can generalize it, shall we say perceptually or conceptually, but I think retrieval really helped in getting the kids able to actually pull it up and, and retrieve it and, and use it. Because without the retrieval, they would not have been as good at that. So I think that's what the retrieval action was, was about for that particular task.

And this is something I hope you don't think is an apples and oranges thing. but… well actually, let me make a different point about the typically developing SLI kind of conditions here. In green, I'm showing you where we found differences. A lot a places we just didn't find group differences, and we had expected group differences. And some of it could be the end, the number of items we taught; totally accept that. I totally accept that. But on the other hand, we tend to think that some, maybe a great part of the word learning weaknesses in these kids might lie elsewhere. We've seen in the literature, people finding good data saying that these kids have difficulties in word encoding. Our design was set up to look at retrieval effects, long term retention effects, and generalization. We were not manipulating encoding. Turns out we have some preliminary evidence within this dataset that is supportive of some encoding weaknesses, and I'd be happy to show you the data if there's time during the questions. But the design wasn't set up to look at the frontend, to look at encoding. But we wanna acknowledge that we were not looking at encoding. We think encoding is really part of the package here.

This is the part where I said I hope you don't think this is an apples to oranges kind of thing. But okay. If, if… something, like a repeated study, is kinda business as usual, say

in a preschool setting or in a home setting where the, the parents, or the preschool teacher might be say reading a, a book with a child, .and introducing the child to new words, okay. That's like sort of repeated study in a naturalistic environment. Or in the case of immediate retrieval, if it's a perfectly natural, and, and okay and positive thing to do where either the, the parent, or in the preschool setting, the teacher turns around and say "Oh yeah, can you say; this, this guy's a giraffe; can you say giraffe?" and actually have the kid immediately repeat the word,. In a nice friendly informal way. If that too is a nice, kind of business, positive, business as usual kind of thing, then consider that that can be done with all kids, right? Then consider the repeated spaced retrieval condition for the SLI kids. 'Cause I'm go—now I'm going over old figures. You've already seen these graphs. But now I'd like you to compare the dark bars to the SLI group, with the lighter gray bars for the typically developing kids. I need you to kind of scan across the whole thing. Dark bars for the SLI, versus the lighter gray bars for the typically developing kids. This is the repeated study condition in the, in the lighter gray bars for the typically developing kids. Business as usual, right? But we're able to get through it the, with the repeated spaced retrieval condition, our SLI kids actually scoring pretty darn high relative to you know repeated study by the typically developing kids.

This next graph is where we were comparing repeated spaced retrieval and immediate retrieval, so number of retrieval opportunities is matched. You know, "Can you say giraffe?" Again, dark bars for the SLI side, versus light gray bars for the typically developing side. And again, a clear edge for repeated spaced retrieval for the kids with SLI.

And now let's take a look at the adjective study. Look at the generalization section here. 'Cause now we're talking about wor—exemplars that were not included in treatment, mkay. The words were, but the kids had to generalize. Dark bar for the SLI kid under generalization, versus, lighter gray bar for the TD kids, under generalization. And once again, you're seeing this advantage for repeated space retrieval. So what we're saying is under optimal conditions, optimal conditions for kids with SLI, we think that we're moving the kids in the right direction if a standard is sort of an experimental version of kind of a business as usual kind of thing for typically developing kids. And we're kinda pleased with that prospect. Now a whole lot a work has to be done to replicate this work, do it under practical conditions to see whether that would hold true. But so far so good.

So next steps. Well, you probably notice that these kids were also not remembering a lot a the words. There are a lot a words these kids didn't know, even if we're getting condition effects. We just need to experiment with more retrieval schedules, determine the optimal number a words, and so on. Then, one a the, our future studies is one where we're asking if words are learned better through repeated spaced retrieval, will the strength of those words be such were it allows them to be better able to do things like adding inflection. So for instance, if we were teaching all the verbs, are the words taught in a repeated spaced retrieval condition, the verbs more likely thereafter to be say, mm, modulated, through inflection? We don't know. This is our next study. And… I haven't; we have a theoretical framework. Because we were not set up, we weren't intending to compare one theoretical count for the other. Our job was, first we need to

establish that retrieval works. But we do have a theoretical framework in one of our next steps, and I'd be happy to talk about it. It, for those of you who are interested, I've got some slides. But… the next step is wanna see whether our assumptions theoretically about what's going on in retrieval are kind of on the mark. 'Cause we know that retrieval is doing what it's doing, but we wanna now ask is the, our assumptions about how it's working and why it's working, are those kind of, kind of accurate. So that's something else that we wanna test in our future work.

So, thank you very much for your attention. (Applause)

Q: Hi, I'm Tiana Cowan from Penn State, I work with Carol Miller. Thank you for a really good talk. I was, I guess curious to hear a little bit more about your theoretical framework, and within that, how you differentiate between rehearsal and retrieval. And I guess, if there's something specific about the motor production of either writing or verbally producing a label that's important for retrieval.

LAURENCE LEONARD: Sure. Sure. Let me answer the second part first before I, uh, then talk a bit about, um… a, um… Maybe I can start with that. First of all, with regard to sort of the motor of the, or the, or the production component. I do think that immediate—we haven't compared immediate retrieval versus repeated study, because we're interested in repeated spaced retrieval, thinking that would hold the advantage. But we don't think that the act of producing the motor aspect of it is playing that big a role in our findings. Because as I was saying, when, in fact, let me, let me actually point to this slide, which I also had, had… holding back in just in case. This is an instance where, in the Experiment Two, we compared repeated spaced retrieval versus immediate retrieval, mkay. And as you recall, for immediate retrieval, the kid sees this is a, "This is a pibe, it's a pibe, it's a pibe," okay. And then the kid gets a retrieval trial, immediately, mkay. That immediate retrieval condition is a very easy condition, because it's almost like just immediate repetition. I mean it's, it's a short time period. The picture goes off, but then it comes back on, it's, I mean you, one could almost argue it's short term phonological memory if you will, if you really wanna be strict about it. Well, as it turns out then, if you look at the IR 1,033, those are the number of times across the kids, where these kids actually during a learning period, they actually produced the word, mkay. In contrast, now almost always, there are a few times when they messed up and they just weren't paying attention and they just didn't say it, but most of the time they said it, it was really a pretty easy condition, during a learning period. For the repeated spaced retrieval, we only had a total of 587 productions during the learning period by these kids. why? Because once you get to the two trial, the first paced trials, you know, just to, to, you know, put words in their mind, it's like might—maybe they were kind of thrown off, like whoa! You know, now you're askin' me to say it, you know, 'cause there had been intervening words in between. So those first few spaced retrials, usually for the kids, even the typically developing kids, these kids end up saying "I don't know." Later on, as, as it accumulated, they started to, to recall these words. So which meant that they had less production practice, because a lot a trials they weren't saying the words at all, whereas in immediate retrieval, they're almost always sayin' the words. And yet, as we saw, the immediate retrieval condition was not as effective in helping retention as

repeated space retrieval. So, so if you just look at using a word, granted, more or less in repetition, it was an immediate retrieval, in response to seeing that picture, it, it is probably more helpful than not saying it, which would occur in the repeated study condition. But it, there's something about the, shall we say the more effort full of spaced retrieval that seems to be beneficial. Now again, we're talkin' about the process of longer, of, of re—retaining something at the end of a second day, and then one week later. And so that's a different process than something that happens immediate. So we think, as I'm sure, a motor factor can be very important, but it wasn't playing a very big role in our project. Back to the, the theory question though. Jeff Karpicke and his colleagues in psychology, um, have used this procedure. For lack of a better term it's a, it's a wordy one, called repeated retrieval with contextual reinstatement. And what basically it, it says that there are contextual features that are out, actually at play here. So an example would be that, and an assumption that they make is when, even in a, in a fairly tightly controlled experimental study, that when one is studying a word, during encoding, there are contextual features, here called Context A, being… associated with the actual item itself. So of course we have some semantic and phonological features and so on. But there are contextual features that are being associated, such that… when retrieval is successful, that context feature gets picked up as well. But then when there's repeated retrieval, and especially when there's spaced retrieval and there are intervening items, the context is changed oh, ever so slightly. And so when there's successful retrieval, that also involves bringing along the original context. Well now you get successful, now you have retrieval in a subsequent trial where the context features are slightly different. We'll call those Context B. So now, and those get added to, to form a composite basically of Context A, Context B, with subsequent retrieval. So here's the spaced retrieval condition. You have Context C being added to it ever so slightly different. So the basic, the sum is that we have these contextual features that with spaced retrieval, where you get little contextual changes, so Context A is not exactly like Context B, not exactly like Context C, but if you then have a composite, the contextual features associated with the item because ever more unique, whereas you're adding slightly different contextual information, therefore the search field if you will, the search set is, is assumed to be reduced, and that seems to be helping. Now, at first, especially when we were doing our task, we kept saying, you know, to Jeff Karpicke, you know, saying you know, that works fine, except it sounds like sure, if you teach, learn the word in this room, and then you go and learn it in this room, the context is changed. We're sayin', we're talkin' about the same crummy picture keeps comin' up. And, um, although the ERP people are really comfortable with that, because when they see these wave forms changing every mm, millisecond, you know, they sort of understand lots of things go on in your brain with, with just a short period of time. But, something that really hit home to me is, when, in an experiment Jeff Karpicke has one, and something we would like to do, applied to, to our SLI kids, is that, there are such things—here, here's an example. This is a case where they're explicitly testing contextual effects. This is a, a study with, with adult learners. They were learning, word lists, mkay, and, the subjects initially were… given, a word list to learn in two sets. There's Set One, and then they learned it, and Set Two was given immediately, and then they had to learn Set Two. Mkay. then there's a little break, and then they would come back. And then they were given, the subjects were given the list as one single list in random order. Now one single list, right?

Now when they're first learning the Set one and Set Two, nothing was singled out to them. They would just had one set, they learned the words, and they're given a second set, learn the words, right? But now they were given a mixed list. One single, all the words are in one set. One, in one condition, the subjects were said just tr—do some more studying, studying this list now, 'cause we're gonna ask you, you know, to remember them later on. People in the other group were said, okay now go over this, and try to remember which set the word was in. Try to remember which set the word was in. And then they got tested. And the people who went through this inter—intervening exercise of trying to remember what word the set was in, did better in the recall of the, of all of the words. But remember, when they were se—presented in separate lists, they weren't told ahead a time, hey, this is in Set One, and this is in Set Two; that's all part of it, you gotta remember that. No. So basically what they're remembering is you wanna call it incidental learning, or just sort of stuff on the side that you happen to pick up. They did, and it affected their recall. So that gave to, in my mind, a lot more credibility to the, the Karpicke notion, that there are things that are not an inherent part of the word itself. None of these individuals thought that, that the contextual detail was part of the word, just as when in the studies that are done with science concepts. No one confused with some little element of the context with the basic science concept. I mean they're, they are somehow kept ja— But it's, it's, it's extra baggage, but in this case, helpful baggage evidently. So anyway that's a longwinded answer to your question.

Q: Thank you.

LAURENCE LEONARD: Thanks for asking it.

Q: Hi, I'm Hannah Krimm. I am an assistant professor at the University of Georgia, and I'm here with Tiffany Hogan. I'm curious about… how feedback lays into the retrieval effect. I know some of Karpicke's other work looks at feedback. And you mentioned that most of these kids either said "I don't know", or they did a correct production. But if they didn't produce the word that you were tryin' to get them to retrieve, what did you guys do? And do you think that feedba—feedback would affect their learning in this, in this context?

LAURENCE LEONARD: Mkay. Yeah, if I understand it, two different things. One is more about the, like the scoring details of the production, and the other was about feedback was it, or?

Q: Yeah, so like if a, if, if you asked a child for… to retrieve one of the words from previous, and they said "I don't know," in the learning condition. **(Oh, oh)** did you, did you just proceed, or did somebody tell them . . .?

LAURENCE LEONARD: No we just, we just proceed, and, and,… yeah, we would just, we would just move on. Oh, we would always get—now, we, as you know, we're, we're always giving encouragement. (Right) You know. So sometimes the kids would, you know, be kinda sad, and we would still—sad in a sense of, you know, on it very good, you know. But you know we'd say "Oh great!" you know. Or you know, always

encouraging the kid. But not differentially. We didn't give differential feedback. 'Cause it-- or did- okay, so . . .

Q: So yes, that was my first question is the clarifying question. And then I'm curious about how since some of their other work has shown that learning with feedback is more effective than learning without fee—or like recall with feedback, is more effective than recall without feedback.

LAURENCE LEONARD: It is, it is. And we did not do, give differential feedback, but although we have not, we have not manipulated that. We've always been consistent across tests. But yeah, from what I know from the, from the retrieval literature with, you know, young adult, uh, par—research participants, and so on, providing feedback- well, let me qualify that. But, in general yes. Providing feedback is helpful. And . . .

Q: So I'm wondering how that squares with the… the difference in the number of retrievals that happen in the immediate retrieval condition versus the spaced retrieval condition.

LAURENCE LEONARD: Uh, uh . . . .

Q: Like it seem—the numbers that you presented was like a thousand and 500 . . .

LAURENCE LEONARD: yeah, yeah . . .

Q: Seem, seems like… it's.. more about something—it's not necessarily about whether they retrieve the word, or whether they retrieve the word correctly. But then how does that square with receiving feedback?

LAURENCE LEONARD: I'm not sure how that squares. (Yeah) The, in the immediate retrieval, I mean, um, they were getting their own feedback, in the sense that if, if they're more likely to repeat it in the immediate retrieval condition, or to the extent that that's self feedback, that, um… that, that could be the case. Where, as was you saw, during the learning period, especially in the early space trials, say, they sometimes didn't come up with a word, and they couldn't therefore, you know, provide feedback to themselves. I, I don't know if you're getting at that aspect of feedback. But if so, didn't a, it didn't do much, because the immediate retrieval condition was not as effective. I, I can say though, that early on, in our, um… in our retrieval trials, early on we had, and in fact, some of those experiments, a study trial followed the retrieval trial. So one could construe that as a type of feedback. Not differential. So for instance in, in a spaced retrieval trial we say, you know, what's this called, what do we call this. A kid doesn't remember that it's a nepp. you know, maybe says "I don't know." "What does it like?" "I don't know." Then a study trial comes up, the same picture of a nepp shows up, and the kid hears, "This is a nepp, it's a nepp, a nepp likes birds." So you can call that feedback. And so we're willing to say, and, and but we always control that if there are, if a, if a study trial followed a retrieval trial, then in the repeated study condition, there are two study trials immediately, you know one right after the other. But, I, I would, I would

claim that that's feedback. It's not differential feedback in the sense that if the kid was right, or the kid was wrong, the kid got that same study trial again, so, but it, so it wasn't differential. But, it, it was a type of feedback. So if a kid was like, almost got it, but just wasn't willing to take a shot at it, and then gets confirmation that maybe the one she was thinking about is… was in fact the right one, that's a type of feedback. And that could of played a role, 'cause we did not manipulate that.

Q: Right, thank you.

LAURENCE LEONARD: Sure.

(Inaudible comment)

Q: Hi, my name is Alicia. I'm a doc student at San Diego State University, slash UC San Diego, and I'm here with my mentor Stephanie De Anda. And, I guess the question that I had was, that you mentioned that you were interested in applying this kind of method to… grammatical morphemes. And, I was wondering what kind of barrier ex— do you expect to see in applying this kind of method, or what might you expect?

LAURENCE LEONARD: Okay. So am I understanding the question? You're referring to in the end when I was talking about next steps? (Yes, um-hm) When I was referring to say if it was novel verbs for example, and… um… and how would we go about, what would we expect to see, or how would we go about testing it, or, like . . .?

Q: Yeah, like what would you expect to see, or, if you might expect any barriers to applying this kind of method?

LAURENCE LEONARD: I don't know what we'd expect to see, is why we, we really did it. But what we would hope for is, well first of all in terms of the, the method we would use, we would be needing to do the verb presentations in say, a, a, either a non-finite context, or in a context where it's say for instance third plural subjects, or something where it's gonna be a bare stem, a zero marked verb form, so the kid doesn't know, and ahead of time, get exposure to the verb, you know, getting inflected in different ways. But, and, and, we, uh, we're not sure we're gonna be doing this study. We need to set it up. And so we have to ask, are we gonna use just activity verbs for that direct object for ease of presentation of animations, or live people performing funny ations. They'll, they'll be novel verbs to be sure. And just teach, teach them as sort of like those activity verbs. But in any case then, after, after the learning period, we would anticipate testing it in such a way where perhaps, we haven't done it yet, but perhaps in a carrier phrase, you know, we would maybe create a habitual context that might obligate a third singular, and say oh look, here we go again, look at this. "Every day the girl…" you know and then she'd be performin' this action, it might be a context, would obligate third singular or something, that's, and maybe test a non-finite verb related inflection like progressive "ing", or maybe a past tense, we don't know yet. But that would be the idea. And so the question is twofold. One of course is the usual; can we just replicate the advantages of repeated spaced retrieval, this time moving to actions as opposed to nouns,

or adjectives which we've already studied? And then second, if so, does that translate into also being more likely to add, in, add something to the verb in the way or a related morpheme and, and then we could possibly break it down into whether it's gonna be a finite morpheme or non-finite mor—morpheme. And so, so that's the general idea. We're hoping it'll be beneficial. We don't think; we're not arguing by the way, that, that that's the best way to teaching finite verb morphology. That's not; the issue is entirely different. The issue is, it does relate in the sense that if you really have a strong verb, that you know you've learned better, are you're just in a better since you know, not all inflections are gonna si—no inflection is gonna simultaneously appear with all verbs at the same time. There are other interacting factors that can influence the, the verb by verb change. But could the strength of the verb representation be one such factor? And would repeated retrie—repeated spaced retrieval help that strength? So.

Q: Thank you.

LAURENCE LEONARD: Thanks for the question. Okay, thank you very much. (Applause)
